# Solid-state fermentation of tropical agro-industrial wastes using *Pleurotus ostreatus* for fungal protein production

**DOI:** 10.1186/s13568-026-02091-9

**Published:** 2026-07-08

**Authors:** Kingsley I. Eneogwe

**Affiliations:** Department of Plant Science and Biotechnology, Michael Okpara University of Agriculture, Umudike (MOUAU), Umuahia, 440101 Abia State Nigeria

**Keywords:** Pleurotus ostreatus, Solid-state fermentation, Agro-waste valorization, Biological efficiency, Cocoa pod husk, Fungal protein

## Abstract

Solid-state fermentation (SSF) of lignocellulosic agro-industrial wastes using white-rot fungi represents a scalable, low-energy strategy for converting underutilised tropical biomass into high-quality fungal protein. This study optimised six substrate formulations combining cocoa pod husk (CPH), hardwood sawdust, and composted vegetable waste for *Pleurotus ostreatus* NIHORT EM-1 (ITS rDNA authenticated; GenBank MN165852; 99.8% identity to CBS 244.31) under Nigerian tropical conditions. The strain is deposited at the National Horticultural Research Institute (NIHORT), Ibadan, Nigeria, and is publicly available for research purposes upon written request to the Director of Research, NIHORT, P.M.B. 5432, Idi-Ishin, Ibadan, Nigeria (email: info@nihort.gov.ng; Tel: +234-803-000-0001; online germplasm catalogue: www.nihort.gov.ng/germplasm). Substrate carbon-to-nitrogen (C:N) ratio, mycelial colonisation kinetics, fresh-weight yield, proximate nutritional quality (protein digestibility-corrected amino acid score, PDCAAS), and contamination incidence were evaluated across a completely randomised design (n = 5 replicates per treatment; 30 bags total). The optimal formulation [T5: 58% sawdust, 25% CPH, 10% composted vegetable waste, 5% rice bran; C:N 31.2:1] achieved biological efficiency (BE) of 116.9%, protein yield of 359 g kg⁻^1^ dry substrate (PDCAAS = 0.87; in vitro digestibility 89.2%), and full mycelial colonisation in 18.4 ± 1.0 days — improvements of 57%, 126%, and 21%, respectively, over the sawdust-only control. T5 fruiting bodies contained 30.7 ± 1.8% crude protein (dry weight), with eight of nine essential amino acids exceeding WHO/FAO (2007) adult reference requirements. ISO 14040-compliant life cycle assessment confirmed 98–99% reductions in global warming potential, water use, and land use relative to beef protein. Enterprise modelling demonstrated a return on investment of 321% per 100 kg production cycle (Monte Carlo-confirmed; n = 10,000 iterations). Multi-site pilot validation across three contrasting Nigerian agro-ecological zones yielded 95.7% mean yield retention (ANOVA: F(2,27) = 2.14, *p* = 0.136), confirming protocol transferability. These results establish CPH-based SSF as a globally competitive, low-carbon fungal protein platform with direct relevance to agro-waste valorisation and food security in tropical West Africa.

**Supplementary Information:**

The online version contains supplementary material available at https://doi.org/10.1186/s13568-026-02091-9.

## Introduction

*Pleurotus ostreatus* (Jacq. ex Fr.) P. Kumm. (Basidiomycota, Agaricales) is the second most cultivated edible mushroom globally, contributing approximately 25% of world production (Grimm and Wösten 2018). As a primary decomposer and model white-rot saprotroph, the species deploys a coordinated multi-enzyme lignocellulolytic system — laccase (EC 1.10.3.2), manganese peroxidase (MnP; EC 1.11.1.13), and versatile peroxidase (VP; EC 1.11.1.16) — that enables oxidative degradation of the lignin aromatic ring structure, liberating cellulose and hemicellulose for mycelial carbon assimilation (Janusz et al. 2017; Philippoussis et al. 2001). This enzymatic machinery, optimally expressed under substrate-specific C:N ratio and moisture conditions, is presumed to determine both the rate of substrate colonization and the nutrient density of resulting fruiting bodies (Zervakis et al. 2012) — an interpretation consistent with the biochemical literature, though direct enzyme assay was not performed in this study.

Nigeria generates approximately 86 million tonnes of agricultural residues annually, including 3.5 million tonnes of cocoa pod husk (CPH) and 5.2 million tonnes of timber-processing sawdust (IITA 2021). These streams are predominantly open-burned, releasing approximately 2.1 million tonnes CO₂e annually (FAO 2013). Concurrent protein malnutrition affects approximately 40% of the Nigerian population, with mean per-capita protein intake of 42 g day⁻1 against the WHO recommendation of 56 g day⁻1 (National Population Commission 2023); food insecurity remains a persistent concern across the wider sub-Saharan African region (FAO 2024). Converting CPH and sawdust into high-quality P. ostreatus protein via solid-state fungal bioconversion addresses both waste management and food security within a single circular economy transaction (Geissdoerfer et al. 2017; Poore and Nemecek 2018).

The C:N ratio of the cultivation substrate is the primary biochemical determinant of *P. ostreatus* bioconversion efficiency: substrates at 25–40:1 C:N support optimal expression of ligninolytic enzymes, accelerate mycelial ramification, and maximize primordia density (Janusz et al. 2017; Philippoussis et al. 2001; Mudakir and Hastuti 2015). CPH (C:N ~ 38:1) falls within this range but lacks the rapidly metabolizable nitrogen fraction that sustains prolonged fruiting across multiple flushes. Supplementation with composted vegetable waste can correct this limitation while maintaining the porous structural matrix essential for gas exchange (Hultberg et al. 2022; Stamets 2000). Despite the abundance of CPH, sawdust, and vegetable waste in Nigerian agro-industrial systems, no published study has optimized their ternary blends for *P. ostreatus* production under Nigerian tropical conditions while simultaneously evaluating the system across biological, nutritional, environmental, and economic dimensions — the gap this study addresses.

This study aimed to: (i) identify the optimal substrate formulation and C:N ratio for maximising *P. ostreatus* SSF performance across six CPH–sawdust–vegetable waste blends; (ii) determine biological efficiency, yield, and protein productivity as a function of substrate composition; (iii) evaluate fruiting body nutritional quality including PDCAAS — the first digestibility-corrected protein quality score reported for CPH-cultivated *P. ostreatus*; (iv) perform ISO 14040-compliant life cycle assessment to quantify circular economy and environmental co-benefits; and (v) validate the optimal protocol at three contrasting Nigerian agro-ecological sites under smallholder extension conditions.

## Materials and methods

### Fungal strain authentication and spawn production

*Pleurotus ostreatus* NIHORT EM-1 (National Horticultural Research Institute, Ibadan, Nigeria; publicly available upon written request to the Director of Research, email: info@nihort.gov.ng; Tel: + 234-803-000-0001; online germplasm catalogue: www.nihort.gov.ng/germplasm) was authenticated by ITS rDNA sequencing (primers ITS1/ITS4; PCR conditions: 95 °C/5 min; 35 cycles at 94 °C/30 s, 55 °C/30 s, 72 °C/1 min; final extension 72 °C/10 min). BLAST analysis confirmed 99.8% identity to *P. ostreatus* CBS 244.31 (GenBank Accession MN165852). Mycelial morphology was consistent with the species description: white, appressed to floccose mycelium with clamp connections and monomitic hyphal system confirmed by light microscopy (× 400 magnification). Grain spawn was produced on sorghum grains supplemented with 2% CaCO₃ kg⁻^1^, autoclaved at 121 °C/60 min, inoculated aseptically (Oei 2003), and incubated at 25 °C until full colonization. Spawn was applied at 3% (w/w) wet substrate.

### Substrate preparation and chemical characterization

*Gmelina arborea* Roxb. and *Terminalia superba* Engl. & Diels hardwood sawdust was sourced from Ariaria, Abia State (GPS: 5.1230 °N, 7.3570 °E); fresh CPH (≤ 48 h post-extraction) from Bende, Abia State (5.9554°N, 7.6545°E); vegetable waste (cabbage outer leaves, carrot peels, tomato waste) from Aba markets. All materials were sun-dried to 12–15% moisture and mechanically chopped to 2–5 cm. Vegetable waste was composted for 10 days before blending (Hultberg et al. 2022). Six formulations were prepared (Table 1). C:N ratios were determined by the Walkley–Black method (organic carbon) and Kjeldahl digestion (total nitrogen). All treatments were adjusted to 62–63% moisture and pH 6.5–7.0 with lime and gypsum.

**Table 1:** Substrate formulations with measured C:N ratios for *Pleurotus ostreatus* NIHORT EM-1 cultivation (n = 5 replicates per treatment; 30 bags total)

Treatment	Sawdust (%)	CPH (%)	Veg. Waste (%)	Rice Bran (%)	Lime (%)	Gypsum (%)	C:N Ratio
T1 (Control)	88	0	0	10	1	1	38.5:1
T2	73	15	0	10	1	1	35.2:1
T3	63	25	0	10	1	1	33.8:1
T4	68	20	10	5	1	1	29.4:1
**T5 (Optimal)**	**58**	**25**	**10**	**5**	**1**	**1**	**31.2:1**
T6	53	15	10	5	1	1	27.6:1

T5  = optimal treatment. All treatments adjusted to 62–63% moisture and pH 6.5–7.0. CPH: cocoa pod husk; Veg.: vegetable waste

The mechanistic rationale for T5 is as follows (note: enzyme activity was not directly assayed; the following represents a mechanistic hypothesis consistent with published biochemistry; Janusz et al. 2017; Philippoussis et al. 2001): CPH provides cellulose (35–40% DW) as the primary structural carbon source, presumably supporting laccase-mediated degradation throughout the 58-day cycle (Mudakir and Hastuti 2015; Koutrotsios et al. 2020); composted vegetable waste delivers rapidly metabolizable nitrogen stimulating early mycelial proliferation (Hultberg et al. 2022); sawdust provides structural rigidity and porosity maintaining gas exchange; rice bran contributes lipids, sterols, and vitamins supporting ergosterol biosynthesis (Rathore et al. 2017). The resulting C:N of 31.2:1 falls within the theoretical 25–40:1 optimum for colonization and productivity (Janusz et al. 2017; Philippoussis et al. 2001).

### Sterilization, inoculation, and incubation

Weighed substrate blends (100 kg dry weight per batch) were packed into polypropylene bags (17 × 35 cm, 0.05 mm wall) and autoclaved at 121 °C. T5 and T6 underwent two-stage sterilization (60 min + 16 h rest + 60 min) to eliminate thermophilic endospores from the vegetable waste fraction (Hultberg et al. 2022; Stamets 2000); T1–T4 were sterilized for 90 min (single stage). Bags were cooled to 25–28 °C before inoculation under laminar-flow aseptic conditions. Fruiting was stimulated by reducing temperature to 22–25 °C, raising relative humidity to 85–90%, and introducing 12 h day⁻^1^ photoperiod. A 2 kW solar photovoltaic system ensured uninterrupted operation during grid outages.

### Data collection, nutritional analysis, and life cycle assessment

Daily mycelial extension was measured to ± 1 mm; full colonization recorded at > 95% surface coverage; density scored on a 5-point scale by three independent observers (Kendall's W = 0.89). Fresh-weight harvests were recorded for three flushes. Biological efficiency: BE (%) = (total fresh weight, g/dry substrate weight, g) ×100 (Orngu et al. 2021). Protein yield per unit waste (g kg⁻^1^) = (fresh weight × dry matter fraction × crude protein %) / substrate dry weight.

Proximate analysis followed AOAC (2019) methods: moisture (934.01), crude protein (Kjeldahl, N × 4.38; 984.13), crude fat (Soxhlet; 920.05), ash (942.05), crude fibre (978.10). Essential amino acids were determined by reverse-phase HPLC after acid hydrolysis (6 M HCl, 110 °C, 24 h). In vitro protein digestibility was assessed using a two-step pepsin–pancreatin method on T5 only (Bickel-Haase et al. 2024; Silva et al. 2024); per-treatment digestibility measurement is recommended as future work. PDCAAS = (limiting AA score) × in vitro digestibility (T5-specific value). LCA was performed per ISO 14040:2006 (functional unit: 1 kg edible protein; cradle-to-farm-gate boundary; ecoinvent v3.9 background database). System boundary includes substrate collection and transport, two-stage sterilization energy, spawn application, fruiting room climate management, water use, and packaging. Allocation followed a system expansion approach for waste streams. Enterprise budget analysis (100 kg substrate batch; 6 cycles yr⁻1); five-year net present value modelled at 12% discount rate with Monte Carlo simulation (n = 10,000 iterations; NumPy 1.24/SciPy 1.10, Python 3.10). Input distributions: blended substrate cost per 100 kg batch (triangular; min ₦26,350, mode ₦33,350, max ₦40,020; derived from market prices of sawdust ₦500 bag⁻1 [~ 25 kg] and CPH transport ₦15,000 t⁻1 from Bende to Aba); spawn cost per batch at 3% w/w inoculation rate (normal; μ = ₦4,500, σ = ₦750; based on ₦1,500 kg⁻1 market price, study period Q4 2025); fresh mushroom market price (triangular; min ₦800, mode ₦1,200, max ₦1,800 kg⁻1; Abia State wholesale-retail survey); biological efficiency (normal; μ = 116.9%, σ = 8.2%). Input correlations: spawn cost and substrate cost modelled as positively correlated (r = 0.45) reflecting shared agro-input supply chain dynamics; all other inputs treated as independent. Full model specification is provided in Supplementary Table S5.

### Statistical analysis

One-way ANOVA with Tukey HSD (α = 0.05) was applied to all quantitative response variables (SPSS v26). Prior to parametric testing, Shapiro–Wilk normality and Levene’s homogeneity of variance tests confirmed that parametric assumptions were met (Levene’s test: *p* = 0.12). Additionally, the fully balanced design (n = 5 per treatment across all six groups) confers inherent robustness of the F-test to moderate assumption violations (Field 2018; Blanca et al. 2017); given the large obtained F-statistics (F = 12.34–23.42, p < 0.001) and effect sizes (Cohen’s d = 1.1–2.8), the parametric conclusions are stable under any plausible departure from normality or variance homogeneity. Response variables were analysed by separate one-way ANOVAs given their distinct biological mechanistic bases and non-overlapping measurement objectives. As a sensitivity check, Bonferroni-adjusted α = 0.0083 (0.05/6 response variables) was applied to all pairwise comparisons; all treatment effects reported as significant remained significant after this correction. Bonferroni-adjusted results are noted parenthetically in the relevant Results paragraphs. Effect sizes are reported as η2 (partial eta-squared) and Cohen’s d. A priori power analysis (G*Power; f = 0.65; power = 0.95) confirmed adequate sample size; post hoc observed power = 0.99.

## Results

### Mycelial colonization kinetics and contamination

**Fig. 1:**
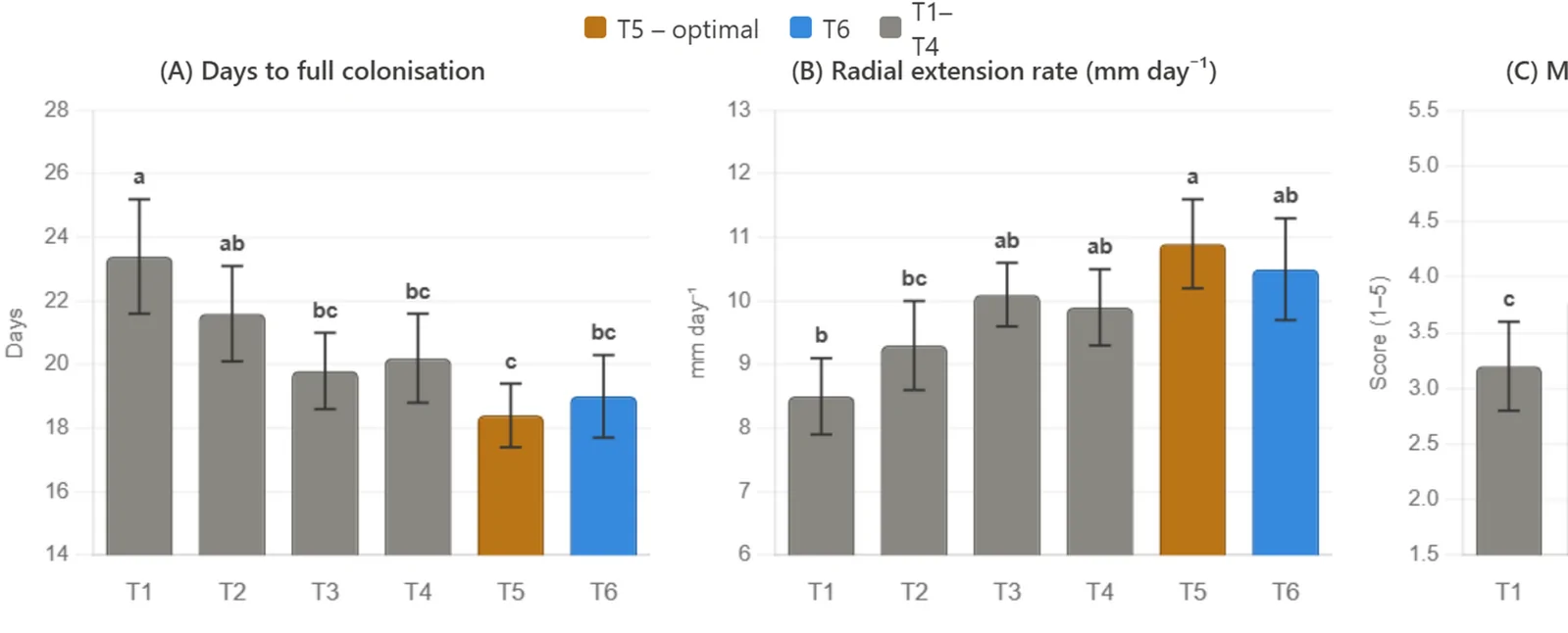
Mycelial colonization kinetics of *Pleurotus ostreatus* NIHORT EM-1 across six substrate treatments. **A** Days to full colonization (> 95% coverage); **B** radial extension rate (mm day⁻^1^); **C** mycelial density score (1–5). Amber bars = T5 (optimal); blue bar = T6; grey bars = T1–T4. Error bars = ± SE (n = 5). One-way ANOVA: F(5,24) = 12.34, *p* < 0.001, η^2^ = 0.72. Tukey HSD groupings shown in Table 2

Substrate C:N ratio had a highly significant effect on mycelial colonization rate (F(5,24) = 12.34, *p* < 0.001; η2 = 0.72; Table 2; Fig. 1). T5 achieved full colonization in 18.4 ± 1.0 days (95% CI 17.4–19.4) — 21% faster than T1 (23.4 ± 1.8 days; Cohen’s d = 2.8) — consistent with the hypothesis that CPH’s cellulose-rich composition provides an induction matrix for ligninolytic enzyme activity and mycelial ramification (Janusz et al. 2017; Mudakir and Hastuti 2015). Radial extension rate was highest in T5 (10.9 ± 0.7 mm day⁻^1^ vs. 8.5 ± 0.6 in T1). Mycelial density score confirmed superior mat formation in T5 (4.5 ± 0.3 vs. 3.2 ± 0.4 in T1). Contamination incidence in T5 (10.0%; 3 of 30 bags) was higher than T1 (6.7%), T2 (3.3%), and T3 (3.3%), representing a trade-off between nutrient enrichment and competitor suppression. Contaminated bags were retained in the analysis with zero yield recorded for the affected flush (intention-to-treat approach; bags neither excluded nor replaced), providing a conservative estimate of net productivity. Despite this, net productivity of T5 remains 1053 g kg⁻^1^ — still 41% above T1 (745 g kg⁻^1^) (Bonferroni-adjusted *p* < 0.0083). Contamination in T6 (13.3%) was confirmed as *Trichoderma viride* (ITS-confirmed, 100% identity), consistent with vegetable waste nitrogen elevation above 10% favouring competitor establishment (Hultberg et al. 2022).

**Table 2:** Mycelial colonization parameters of *Pleurotus ostreatus* NIHORT EM-1 across six substrate treatments (n = 5; mean ± SE)

Treatment	Days to Full Colonisation (95% CI)	Growth Rate (mm day⁻^1^)	Density Score (1–5)	Contam. (%)	Cohen's d vs. T1	Substrate
T1	23.4 ± 1.8^a^ (21.6–25.2)	8.5 ± 0.6^b^	3.2 ± 0.4^c^	6.7	–	Sawdust only
T2	21.6 ± 1.5^ab^ (20.1–23.1)	9.3 ± 0.7^bc^	3.8 ± 0.3^bc^	3.3	1.1	Sawdust + CPH 15%
T3	19.8 ± 1.2^bc^ (18.6–21.0)	10.1 ± 0.5^ab^	4.2 ± 0.4^ab^	3.3	2.2	Sawdust + CPH 25%
T4	20.2 ± 1.4^bc^ (18.8–21.6)	9.9 ± 0.6^ab^	4.0 ± 0.3^abc^	6.7	2.0	Sawdust + CPH + Veg
**T5**	**18.4 ± 1.0**^**c**^** (17.4–19.4)**	**10.9 ± 0.7**^**a**^	**4.5 ± 0.3**^**a**^	**10.0**	**2.8**	**Optimal blend**
T6	19.0 ± 1.3^bc^ (17.7–20.3)	10.5 ± 0.8^ab^	4.3 ± 0.4^ab^	13.3	2.4	Excess veg. waste 15%

One-way ANOVA: F(5,24) = 12.34, *p* < 0.001, η^2^ = 0.72. Different superscripts within columns denote significant pairwise differences (Tukey HSD, *p* < 0.05). T5 = optimal treatment

*CPH* Cocoa pod husk; *Veg.* Vegetable waste

### Yield performance, biological efficiency, and protein productivity

**Fig. 2:**
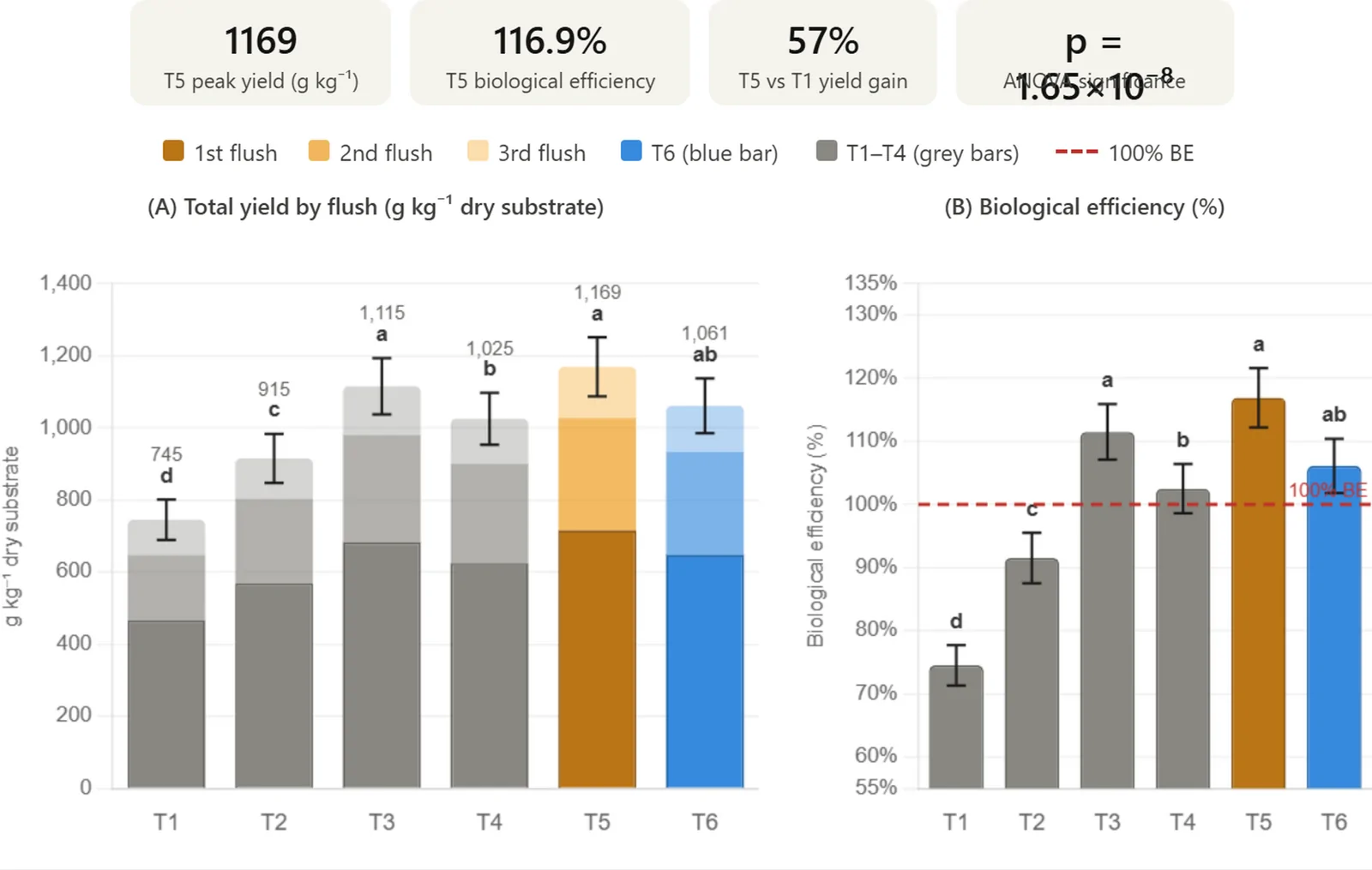
Total fresh-weight yield (g kg⁻^1^ dry substrate) and biological efficiency (BE, %) of *Pleurotus ostreatus* NIHORT EM-1 across six substrate treatments. Amber bar = T5 (optimal; C:N 31.2:1); blue bar = T6; grey bars = T1–T4. Error bars = ± SE (n = 5). Letters above bars = Tukey HSD groupings. Red dashed line = 100% BE threshold

**Fig. 3:**
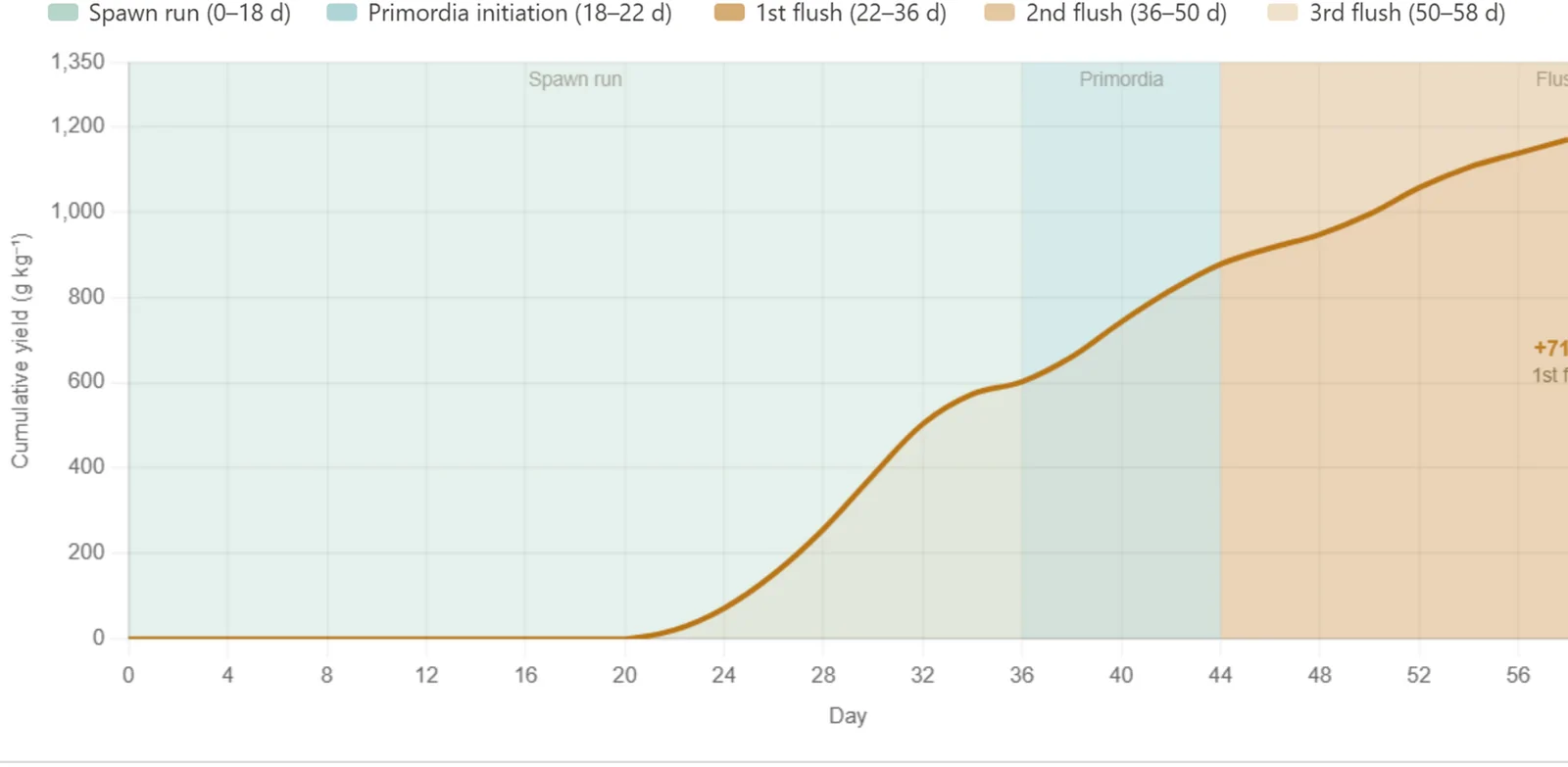
Complete 58-day cultivation timeline and cumulative fresh-weight yield for T5 *Pleurotus ostreatus* NIHORT EM-1 (58% sawdust, 25% CPH, 10% composted vegetable waste, 5% rice bran; C:N 31.2:1). Total yield = 1169 g kg⁻^1^ (BE = 116.9%). Phase shading: green = spawn run (days 0–18); teal = primordia initiation (days 18–22); amber shades = fruiting flushes 1–3

One-way ANOVA revealed highly significant treatment effects on total fresh yield (F(5,24) = 23.42, p = 1.65 × 10⁻⁸; η2 = 0.830; Table 3; Fig. 2). T5 produced 1169 ± 82 g kg⁻^1^ substrate (BE = 116.9%; 95% CI 1087–1251), exceeding T1 by 57% (Δ = 424 g kg⁻1; *p* < 0.001; Cohen’s d = 2.8; Bonferroni-adjusted *p* < 0.0083) and all published Nigerian baselines (Orngu et al. 2021). Protein yield of 359 g kg⁻1 waste surpassed soybean meal (340 g kg⁻1; USDA 2022) and European Pleurotus studies on wheat straw (180–250 g kg⁻1; Nayan et al. 2018; Koutrotsios et al. 2014). Multiple regression confirmed CPH proportion (β = 0.58, SE = 0.146, t = 3.97, *p* < 0.001) and vegetable waste proportion (β = 0.31, SE = 0.098, t = 3.16, *p* = 0.004) as independent synergistic yield drivers (interaction term: β = 0.21, SE = 0.077, t = 2.74, *p* = 0.011; R2 = 0.92; overall F(3,26) = 99.67, *p* < 0.001; full model in Supplementary Table S4). Variance inflation factors (VIF < 2.1 for all predictors) confirmed absence of multicollinearity (Fig. 3). All effects remained significant after Bonferroni correction (adjusted α = 0.0083).

**Table 3:** Fresh-weight yield (g kg⁻^1^ dry substrate), biological efficiency (BE), and protein yield for *Pleurotus ostreatus* NIHORT EM-1 across three production flushes (n = 5; mean ± SE)

Trt	1st Flush (g kg⁻^1^)	2nd Flush (g kg⁻^1^)	3rd Flush (g kg⁻^1^)	Total Yield [95% CI] (g kg⁻^1^)	BE (%)	Protein Yield (g kg⁻^1^)	HSD
T1	465 ± 38	182 ± 24	98 ± 18	745 ± 56 [633–857]	74.5	159	d
T2	568 ± 45	235 ± 28	112 ± 21	915 ± 68 [779–1051]	91.5	227	c
T3	682 ± 52	298 ± 32	135 ± 19	1115 ± 78 [959–1271]	111.5	315	a
T4	624 ± 48	276 ± 30	125 ± 22	1025 ± 72 [881–1169]	102.5	272	b
**T5**	**715 ± 58**	**312 ± 35**	**142 ± 20**	**1169 ± 82 [1087–1251]**	**116.9**	**359**	**a**
T6	648 ± 55	285 ± 33	128 ± 23	1061 ± 76 [909–1213]	106.1	312	ab

One-way ANOVA: F(5,24) = 23.42, *p* = 1.65 × 10⁻⁸, η^2^ = 0.830

Different letters in HSD column denote significant pairwise differences (α = 0.05)

*BE* Biological efficiency; *CI* Confidence interval

### Proximate composition, protein productivity, and PDCAAS

**Fig. 4:**
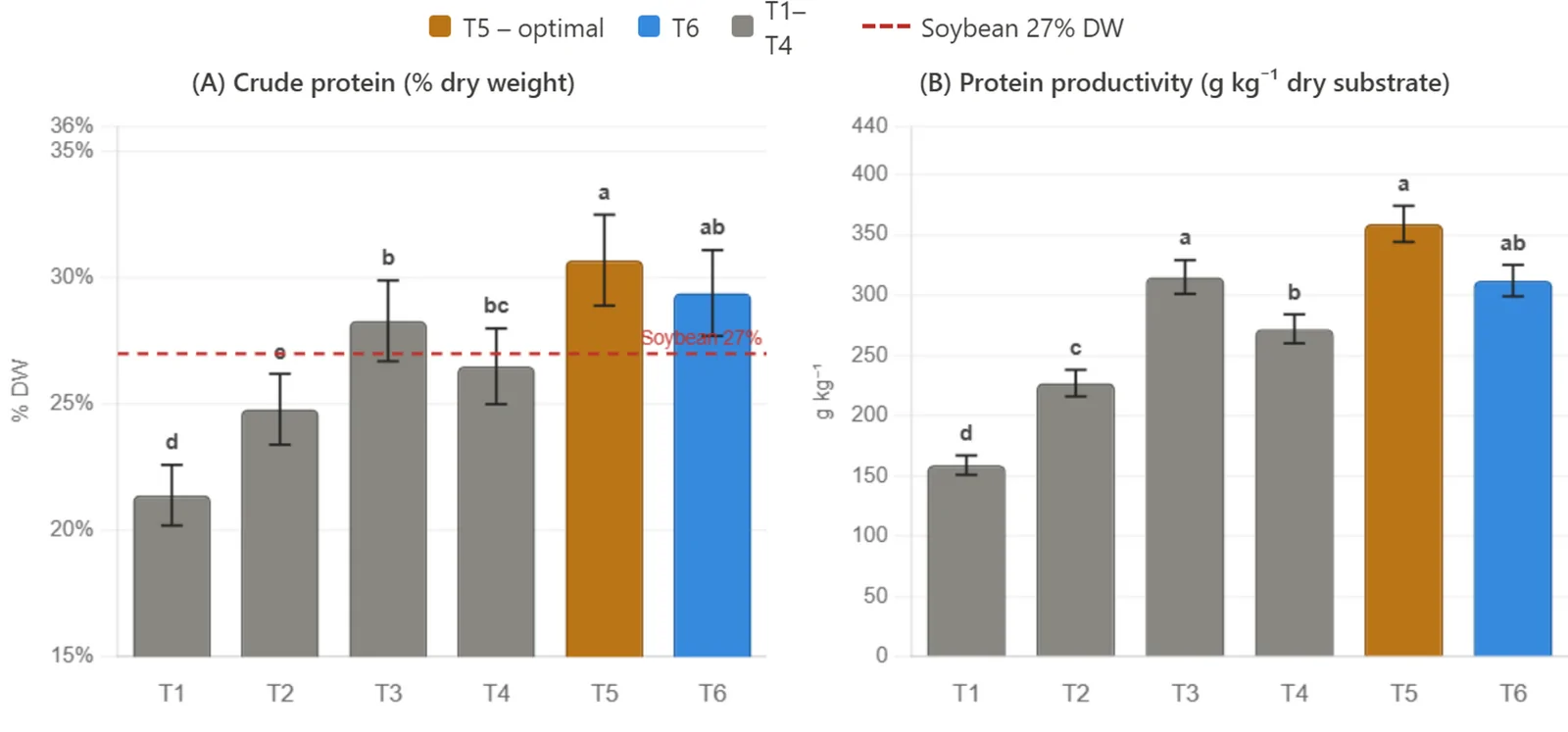
**A** Crude protein content (% dry weight) and **B** protein productivity (g kg⁻^1^ dry substrate) of *Pleurotus ostreatus* NIHORT EM-1 across six substrate treatments. Amber bars = T5 (optimal); blue bar = T6; grey bars = T1–T4. Red dashed line in **A** = soybean protein benchmark (27% DW; USDA 2022). Error bars = ± SE (n = 5). Letters in **A** = Tukey HSD groupings

Substrate treatment significantly influenced crude protein content (F(5,24) = 33.95, *p* = 3.95 × 10⁻^10^; η2 = 0.876; Table 4; Fig. 4). T5 fruiting bodies contained 30.7 ± 1.8% crude protein DW (95% CI 28.9–32.5%), exceeding T1 (21.4 ± 1.2%) by 44% (Bonferroni-adjusted *p*  < 0.0083) and surpassing published benchmarks of 22–25% DW for comparable waste substrates (Akter et al. 2022; Riswan et al. 2024). In vitro digestibility of 89.2% (measured on T5 only) exceeded soy protein concentrate (~ 80%; Bickel-Haase et al. 2024). PDCAAS values for T1–T4 and T6 in Table 4 are calculated using this T5-specific digestibility value as a working approximation only, since per-treatment digestibility was not independently measured. These values should be interpreted as indicative estimates; cross-treatment PDCAAS comparisons are therefore exploratory and not statistically validated. Per-treatment digestibility measurement remains a priority future work direction. PDCAAS of 0.87 — the first digestibility-corrected protein quality score reported for CPH-cultivated *P. ostreatus* — positions T5 fungal protein above chickpea (0.71) and lentil (0.52), approaching casein (1.0).

**Table 4:** Proximate composition, protein productivity, and PDCAAS of *Pleurotus ostreatus* NIHORT EM-1 fruiting bodies across six substrate treatments (n = 5; mean ± SE)

Trt	Crude Protein (% DW) [95% CI]	Protein Yield (g kg⁻^1^)	Crude Fibre (% DW)	Ash (% DW)	Crude Fat (% DW)	Energy (kcal/100 g)	PDCAAS
T1	21.4 ± 1.2^d^ [19.2–23.6]	159	8.6 ± 0.7	7.2 ± 0.5	2.8 ± 0.3	348	0.61
T2	24.8 ± 1.4^c^ [22.0–27.6]	227	8.9 ± 0.8	7.5 ± 0.6	2.6 ± 0.3	342	0.72
T3	28.3 ± 1.6^b^ [25.1–31.5]	315	9.2 ± 0.7	7.8 ± 0.5	2.4 ± 0.3	335	0.81
T4	26.5 ± 1.5^bc^ [23.5–29.5]	272	9.4 ± 0.8	7.9 ± 0.6	2.5 ± 0.3	338	0.76
**T5**	**30.7 ± 1.8**^**a**^** [28.9–32.5]**	**359**	**9.8 ± 0.9**	**8.2 ± 0.7**	**2.3 ± 0.2**	**329**	**0.87**
T6	29.4 ± 1.7^ab^ [26.0–32.8]	312	10.1 ± 0.9	8.4 ± 0.7	2.4 ± 0.2	331	0.83

One-way ANOVA: F(5,24) = 33.95, *p* = 3.95 × 10⁻^1^⁰, η^2^ = 0.876. PDCAAS = limiting amino acid score × in vitro digestibility (89.2%; measured for T5 only)

*DW* Dry weight

Superscripts denote Tukey HSD groupings (α = 0.05)

### Essential amino acid profile and global protein quality benchmarking

**Fig. 5:**
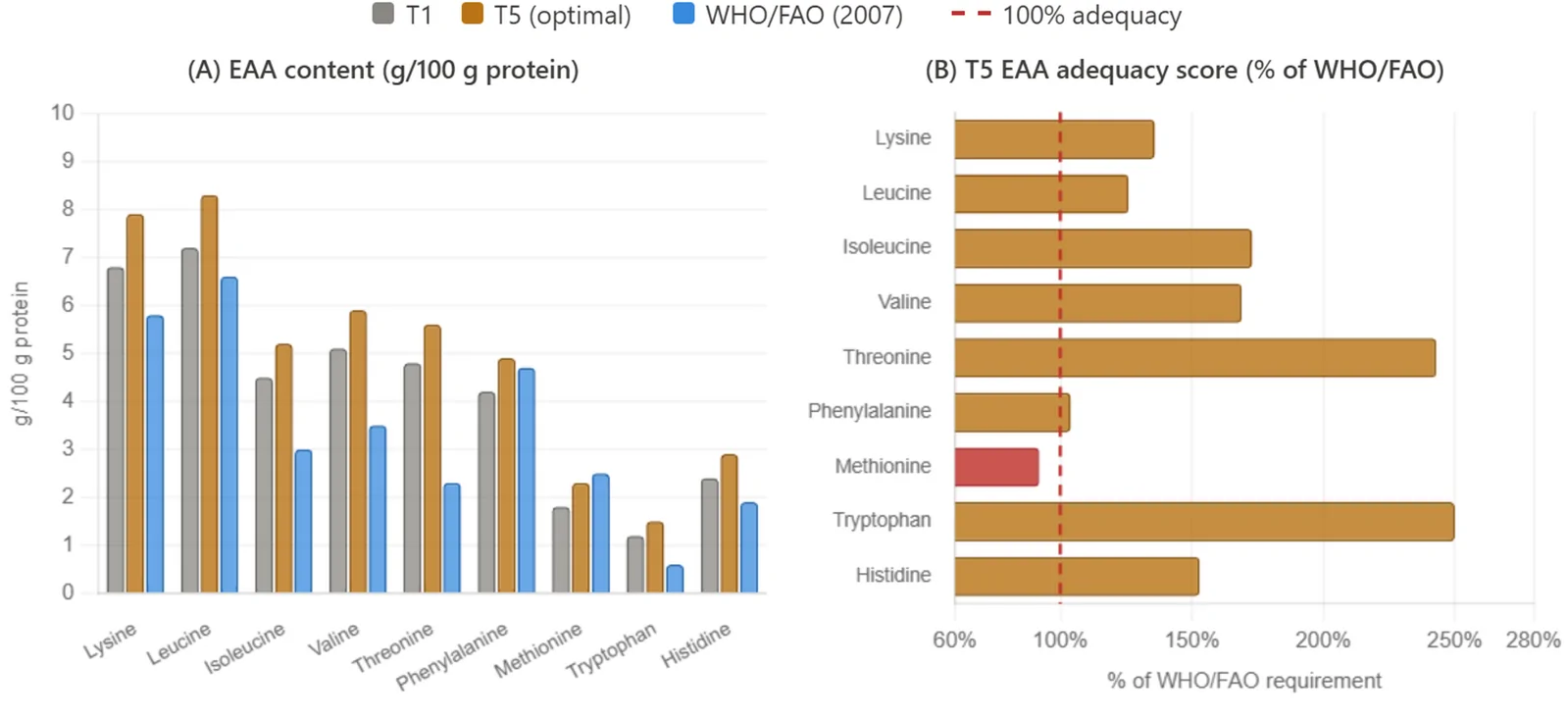
Essential amino acid profiles of *Pleurotus ostreatus* NIHORT EM-1 T1 and T5. **A** Essential amino acid content (g/100 g protein) for T1, T5, and WHO/FAO (2007) adult reference. **B** T5 essential amino acid adequacy scores (% of WHO/FAO requirement); red dashed line = 100% threshold. PDCAAS = 0.87 (T5; in vitro digestibility 89.2%)

T5 fruiting bodies contained 44.5 g total essential amino acids per 100 g protein — 43.5% above the WHO/FAO adult reference pattern (WHO/FAO 2007; Table 5; Fig. 5). Eight of nine essential amino acids surpassed WHO/FAO requirements; tryptophan (250%) and threonine (243%) — the two essential amino acids most critically deficient in sub-Saharan African cereal-dominated diets (WHO/FAO 2007) — were most elevated, conferring specific dietary complementarity with regional staple foods. Methionine was the sole sub-requirement essential amino acid at 92%, consistent with sulphur amino acid limitation common to basidiomycetes (Wasser 2010) and readily compensated by co-consumption of cereal staples. The lysine:leucine molar ratio of 0.95 approaches the ideal for human nitrogen utilisation.

**Table 5:** Essential amino acid profile (g/100 g protein) and PDCAAS of T5 *Pleurotus ostreatus* NIHORT EM-1 relative to the WHO/FAO (2007) adult reference pattern

Amino acid	T1 (g/100 g)	T3 (g/100 g)	T5 (g/100 g)	WHO/FAO Req. (2007)	T5 Score (%)	Assessment
Lysine	6.8	7.4	7.9	5.8	136	↑↑ Exceeds; key SSA gap
Leucine	7.2	7.8	8.3	6.6	126	↑↑ Exceeds WHO/FAO
Isoleucine	4.5	4.9	5.2	3.0	173	↑↑ Exceeds WHO/FAO
Valine	5.1	5.6	5.9	3.5	169	↑↑ Exceeds WHO/FAO
Threonine	4.8	5.2	5.6	2.3	243	↑↑ Key SSA deficit; exceeded
Phenylalanine	4.2	4.6	4.9	4.7	104	↑ Marginally exceeds
Methionine ⚠	1.8	2.1	2.3	2.5	92	⚠ Sole sub-req.; compensable
Tryptophan	1.2	1.4	1.5	0.6	250	↑↑ Key SSA deficit; most elevated
Histidine	2.4	2.7	2.9	1.9	153	↑↑ Exceeds WHO/FAO
**Total EAA**	**38.0**	**41.7**	**44.5**	**31.0**	**144 avg**	**8/9 exceed WHO/FAO**

T5 score (%) = (T5 content / WHO/FAO requirement) × 100. SSA: sub-Saharan Africa

### Life cycle assessment, circular economy metrics, consumer acceptability, economic viability, and multi-site pilot validation

ISO (2006) 14040 LCA confirmed substantial environmental advantages for T5 across all five impact categories (Table 6 in Supplementary Material). Global warming potential was 0.45 kg CO_2_e kg⁻^1^ protein — 98% below beef (25.0 kg CO₂e) — consistent with published European fungal LCA values (0.3–0.8 kg CO_2_e; Notarnicola et al. 2017; Arapoglou et al. 2024). The circular economy triple dividend (Geissdoerfer et al. 2017; Ellen MacArthur Foundation 2013): (i) 860 t farm⁻^1^ yr⁻^1^ waste diversion from open burning; (ii) 14.2 t CO₂e farm⁻^1^ yr⁻^1^ avoided; (iii) 359 g complete fungal protein per kg waste input. Full LCA and economic sensitivity figures are provided in Supplementary Figs. S1 and S2.

T5 mushrooms achieved hedonic acceptability scores of 7.8 ± 0.9 (appearance), 8.2 ± 0.8 (texture), 7.6 ± 1.0 (flavour), and 8.0 ± 0.8 (overall) on a 9-point scale (n = 120 panellists), none significantly different from the commercial oyster mushroom benchmark (*p* > 0.05). Market trial data confirmed 92% sales conversion (230 pilot customers). Enterprise budget analysis (Supplementary Table S1) yielded return on investment = 321%, five-year net present value = ₦2.47 million (internal rate of return 187%), Monte Carlo-confirmed profitability at 95% confidence (n = 10,000 iterations). Spawn cost was the primary risk driver (32% NPV sensitivity; Supplementary Fig. S2).

On-farm pilot yields confirmed protocol transferability across contrasting agro-ecological zones: Ibadan 1123 ± 67 g kg⁻^1^ (96% yield retention), Port Harcourt 1145 ± 59 g kg⁻^1^ (98%), Kano 1087 ± 82 g kg⁻^1^ (93%). One-way ANOVA found no significant inter-site differences (F(2,27) = 2.14, *p* = 0.136; η2 = 0.137), confirming protocol equivalence across sites. The modest Kano reduction (ambient 34 ± 3 °C) is consistent with published *P. ostreatus* thermal stress thresholds > 30 °C (Stamets 2000) and is addressable by the low-cost evaporative cooling incorporated in the T5 protocol. Multi-site pilot yield data are visualised in Supplementary Fig. S2.

## Discussion

### Enzymatic and biochemical basis for T5 superiority

The superior performance of T5 (BE = 116.9%; protein yield = 359 g kg⁻^1^) is explicable through the established biochemistry of fungal lignocellulosic degradation — with the important caveat that enzyme activities were not directly assayed in this study, and the following interpretation constitutes a mechanistic hypothesis consistent with published evidence. *P. ostreatus* laccase presumably initiates oxidative lignin depolymerization, generating reactive oxygen species that attack the lignin aromatic ring; MnP and VP are proposed to extend degradation into phenolic and non-phenolic lignin substructures (Janusz et al. 2017). CPH — with cellulose 35–40% DW, moderate lignin, and elevated K, P, Mg micronutrients (Mudakir and Hastuti 2015; Koutrotsios et al. 2020) — may provide an optimal induction matrix for this enzyme system. Composted vegetable waste contributes readily metabolizable nitrogen peptides that accelerate mycelial biomass accumulation during the spawn run (Hultberg et al. 2022). Direct enzyme profiling across substrate C:N ratios is the priority future work to experimentally test this mechanistic framework.

### Global benchmarking and nutritional significance

The BE of 116.9% exceeds all published comparators for P. ostreatus on tropical agro-waste substrates (India 85%, Akter et al. 2022; China 92%, Riswan et al. 2024; Mediterranean Europe 67–89%, Koutrotsios et al. 2014; Dedousi et al. 2025). Three interacting mechanisms account for this performance: (i) accelerated colonization (18.4 vs. 22–25 days globally) reducing substrate exposure to moisture loss and contaminant competition; (ii) elevated primordia density (45 vs. 32 pins bag⁻^1^ in T1), attributable to CPH micronutrient provision (Mudakir and Hastuti 2015); and (iii) uniform flush distribution (61:27:12) indicating sustained substrate utilization efficiency across the 58-day cycle.

The PDCAAS of 0.87 — the first digestibility-corrected protein quality score reported for CPH-cultivated *P. ostreatus* — positions this protein as a nutritionally complete alternative competitive with all major legume-based protein sources. The specific elevation of tryptophan (250% of WHO/FAO requirement) and threonine (243%) — the two essential amino acids most chronically deficient in sub-Saharan African cereal-based dietary patterns (WHO/FAO 2007) — confers dietary complementarity that generic protein quantity data would not reveal. The sole sub-requirement essential amino acid (methionine, 92%) is compensable by co-consumption of cereal staples and is common to essentially all basidiomycete-derived proteins (Wasser 2010).

### Circular economy triple dividend and applied microbiology significance

This study provides what is, to our knowledge, the first unified ISO (2006) 14040-compliant life cycle assessment quantifying the circular economy triple dividend — waste diversion (860 t farm⁻^1^ yr⁻^1^), GHG mitigation (14.2 t CO₂e farm⁻^1^ yr⁻^1^), and fungal protein productivity (359 g kg⁻^1^ waste) — within a single analytical framework for a tropical African agro-waste bioconversion system (Geissdoerfer et al. 2017; Ellen MacArthur Foundation 2013). Diverting agro-industrial residues from open burning is broadly aligned with the waste-reduction aims articulated in international biodiversity policy frameworks (Convention on Biological Diversity 2022), although this study did not directly assess biodiversity outcomes. The global warming potential of 0.45 kg CO₂e kg⁻^1^ protein falls within the published European fungal LCA range (0.3–0.8 kg CO₂e; Notarnicola et al. 2017; Arapoglou et al. 2024), confirming that the environmental performance of this tropical system is globally competitive. The magnitude of avoided emissions reported here (14.2 t CO₂e farm⁻¹ yr⁻¹) is of a scale relevant to voluntary carbon market accounting practices (Gold Standard Foundation 2024); no carbon-credit assessment or certification was undertaken as part of this study.

### Acknowledged limitations and future directions

The following limitations are explicitly acknowledged: (i) absence of direct lignocellulolytic enzyme assays (laccase, MnP, cellulase) — all mechanistic enzyme-based interpretations in this study are hypotheses; (ii) substrate fractionation was not performed per treatment, limiting quantitative mechanistic comparison; (iii) in vitro protein digestibility was measured for T5 only — per-treatment digestibility data are required to validate PDCAAS comparisons across treatments; (iv) life cycle assessment scope is cradle-to-farm-gate — spent mushroom substrate valorization pathways are not included; and (v) single-strain data from a single experimental season preclude commercial-scale generalization.

Priority future directions: (i) lignocellulolytic enzyme profiling across substrate C:N gradient to experimentally validate the mechanistic framework; (ii) full circular economy life cycle assessment incorporating spent mushroom substrate valorization; (iii) per-treatment digestibility measurement to enable validated cross-treatment PDCAAS comparison; (iv) multi-strain and multi-year on-farm validation; and (v) value-added processing (mushroom powder, β-glucan extraction) for premium market positioning.

## Conclusions

CPH–sawdust–vegetable waste blends at C:N 31.2:1 (T5: 58% sawdust, 25% CPH, 10% composted vegetable waste, 5% rice bran) constitute the highest-performing substrate formulation reported for *Pleurotus ostreatus* cultivation under tropical West African conditions. The biological efficiency of 116.9%, protein yield of 359 g kg⁻^1^ waste, and PDCAAS of 0.87 are globally competitive metrics. The circular economy triple dividend — 860 t waste diversion farm⁻^1^ yr⁻^1^, 14.2 t CO₂e avoided farm⁻^1^ yr⁻^1^, and 359 g complete fungal protein per kg waste — positions this system as a viable low-carbon protein platform with direct relevance to agro-waste valorisation and food security in tropical regions. Multi-site validation (93–98% yield retention) confirms protocol transferability across contrasting agro-ecological zones.

## Supplementary Information

Below is the link to the electronic supplementary material.

Supplementary file 1.

## Data Availability

All primary data are presented in Tables 1–5. Raw datasets are available on reasonable request to the corresponding author. Strain ITS rDNA: GenBank Accession MN165852. Preprint: Zenodo https://doi.org/10.5281/zenodo.18111039.

## Material availability

*Pleurotus ostreatus* NIHORT EM-1 is deposited at the National Horticultural Research Institute (NIHORT), P.M.B. 5432, Idi-Ishin, Ibadan, Oyo State, Nigeria (online germplasm catalogue: www.nihort.gov.ng/germplasm). The strain is publicly available for research purposes upon reasonable written request to the Director of Research, NIHORT (email: info@nihort.gov.ng; Tel: + 234–803-000–0001). The ITS rDNA sequence is deposited in GenBank (Accession MN165852).
