# Supplementary material for: Solid-state fermentation of tropical agro-industrial wastes using Pleurotus ostreatus for fungal protein production
Source: AMB Express. 2026 Jul 8;16:99. doi: 10.1186/s13568-026-02091-9 (PMC13631136; doi:10.1186/s13568-026-02091-9)
Supplement: Supplementary file 1 [file 13568_2026_2091_MOESM1_ESM.docx]

## SUPPLEMENTARY MATERIAL

## Table S1. Enterprise Budget for a Single 100 kg T5 Pleurotus ostreatus NIHORT EM-1 Production Batch (Q4 2025 Naira Prices; n = 5 Replicate Batches Verified)

| **Cost Item** | **Cost (₦)** | **% Total** | **Qty** | **Unit Cost (₦)** | **Category** | **Remark** |
| --- | --- | --- | --- | --- | --- | --- |
| Sawdust (58 kg) | 2,900 | 8.7 | 58 kg | ₦50/kg | Raw material | Locally sourced; zero pre-treatment |
| Cocoa pod husk (25 kg) | 1,250 | 3.7 | 25 kg | ₦50/kg | Raw material | Farm-gate collection |
| Vegetable waste (10 kg) | 0 | 0.0 | 10 kg | ₦0 | Raw material | Market waste; zero cost |
| Rice bran (5 kg) | 1,500 | 4.5 | 5 kg | ₦300/kg | Supplement | N-supplement; ergosterol precursor [28] |
| Lime + gypsum (2 kg ea.) | 400 | 1.2 | 4 kg | ₦100/kg | Amendment | pH buffer; structural agent |
| Spawn – NIHORT EM-1 (3 kg) | 4,500 | 13.5 | 3 kg | ₦1,500/kg | Biological | Primary NPV risk driver (32% sensitivity) |
| Packaging materials | 3,000 | 9.0 | 30 bags | ₦100/bag | Equipment | Reusable 2–3 cycles |
| Energy | 5,500 | 16.5 | — | — | Overhead | Solar PV backup; 2 kW system |
| Labour (2 workers, 10 days) | 8,000 | 24.0 | 20 p-days | ₦400/day | Labour | 70% women/youth in pilot farms |
| Water | 800 | 2.4 | ~12,000 L | — | Overhead | 120 L kg⁻¹ protein |
| Infrastructure (depreciated) | 5,500 | 16.5 | — | — | Capital | Depreciated over 6 cycles yr⁻¹ |
| TOTAL | 33,350 | 100.0 | — | — | — | Per 100 kg dry substrate batch |

Revenue: wholesale ₦93,520; retail ₦140,280. ROI = (Revenue − Cost)/Cost × 100 = 321%. Annual revenue per farm (6 cycles yr⁻¹) = ₦642,000. Five-year NPV = ₦2.47 million (IRR = 187%; 12% discount rate). Monte Carlo simulation (n = 10,000 iterations) confirmed P(profit) > 95%. Spawn cost is the primary NPV risk driver (32% sensitivity). p-days: person-days.
